# Qualitative Analysis of Daily Diary Response Data From Hispanic and Latino ADRD Family Caregivers

**DOI:** 10.1093/geroni/igaf122.1705

**Published:** 2025-12-31

**Authors:** Stephanie DiFiglia, Elisha Underwood, Sarah Khalidi, Loreli Alvarez, Michael Crowe, Frank Puga

**Affiliations:** Metropolitan Jewish Health System, Inc. Institute for Innovation in Palliative Care, New York, New York, United States; The University of Alabama at Birmingham, Birmingham, Alabama, United States; The University of Alabama at Birmingham, Birmingham, Alabama, United States; The University of Alabama at Birmingham, Birmingham, Alabama, United States; University of Alabama at Birmingham, Birmingham, Alabama, United States; University of Alabama at Birmingham, Birmingham, Alabama, United States

## Abstract

Hispanic and Latino (H&L) family caregivers of individuals living with dementia experience worse mental health outcomes than non-caregiver peers. The Nuestros Dias (Our Days) study examines multi-level factors influencing their daily and distal mental health outcomes using a multi-wave assessment. Eligible H&L caregivers were >18 years who provide unpaid care to a family member living with dementia (assisting with >1 Activity of Daily Living [ADL] or > 2 Instrumental ADLs). The purpose of this study was to examine preliminary themes emerging from participants’ qualitative responses to the open-ended question, “Did anything else happen today?”. This question was provided in each Wave 1 daily diary survey for 21 days. Participants included 11 caregivers who completed >1 open-ended response via REDCap. From the 231 daily diary surveys sent to the 11 participants, 88 qualitative responses were recorded. Two coders independently coded 88 responses, reaching consensus with a third coder. Participants had an average age of 55.2 years; most were female (n = 10), and half (n = 5) were of Mexican background. Most provided care to a parent (n = 7). Preliminary themes included emotional challenges of providing care (n = 10 participants), engagement/support from family/friends/formal caregivers (n = 5), engagement in wellness/self-care activities (n = 4), and struggles managing dementia-related behaviors (n = 4), as well as positive daily experiences (n = 6). Initial data show that H&L caregivers report both positive and negative caregiving experiences, with unmet needs in managing challenging behaviors and emotional distress. These findings provide early insights into the daily experiences of H&L caregivers that can inform culturally responsive interventions to support mental health.

